# Association between Inflammatory Markers and Local Recurrence in Patients with Giant Cell Tumor of Bone: A Preliminary Result

**DOI:** 10.3390/curroncol30010085

**Published:** 2023-01-13

**Authors:** Shinji Tsukamoto, Andreas F. Mavrogenis, Rebeca Angulo Alvarado, Matteo Traversari, Manabu Akahane, Kanya Honoki, Yasuhito Tanaka, Davide Maria Donati, Costantino Errani

**Affiliations:** 1 Department of Orthopaedic Surgery, Nara Medical University, 840, Shijo-cho, Kashihara 634-8521, Nara, Japan; 2 First Department of Orthopaedics, National and Kapodistrian University of Athens, School of Medicine,41 Ventouri Street, 15562 Athens, Greece; 3 Department of Orthopaedic Oncology, IRCCS Istituto Ortopedico Rizzoli, Via Pupilli 1, 40136 Bologna, Italy; 4 Department of Health and Welfare Services, National Institute of Public Health, 2-3-6 Minami, Wako-shi 351-0197, Saitama, Japan

**Keywords:** giant cell tumor of bone, neutrophil-lymphocyte ratio, modified Glasgow prognostic score, prognostic nutrient index, lymphocyte-monocyte ratio, platelet-lymphocyte ratio, hemoglobin, alkaline phosphatase, lactate dehydrogenase

## Abstract

Giant cell tumor of bone (GCTB) has a high local recurrence rate of approximately 20%. Systemic inflammatory markers, such as neutrophil-lymphocyte ratio (NLR), modified Glasgow prognostic score (mGPS), prognostic nutritional index (PNI), lymphocyte-monocyte ratio (LMR), platelet-lymphocyte ratio (PLR), hemoglobin (Hb), alkaline phosphatase (ALP), and lactate dehydrogenase (LDH), have been reported as prognostic markers in patients with malignant tumors. This study aimed to investigate the correlation between these markers and the local recurrence rate of GCTB. In total, 103 patients with GCTB who underwent surgery at the authors’ institutions between 1993 and 2021 were included. Thirty patients experienced local recurrence. Univariate and multivariate analysis showed that tumor site, preoperative and postoperative denosumab treatment, and surgery were significantly associated with local recurrence-free survival. LDH was associated with local recurrence-free survival on univariate analysis only. NLR, mGPS, PNI, LMR, and PLR score did not correlate with the local recurrence rate. In conclusion, NLR, mGPS, PNI, LMR, PLR score, Hb, ALP, and LDH levels are not correlated with the local recurrence rate of GCTB. However, due to the small number of patients included in this study, this result should be re-evaluated in a multicenter study with a larger sample size.

## 1. Introduction

Giant cell tumor of bone (GCTB) is an intermediate-grade primary bone tumor that accounts for approximately 5% of primary bone tumors [1]. The predilection age of GCTB is approximately 30 years [1]. The distal femur (30%) is the most common site of GCTB, followed by the proximal tibia (28%), distal radius (9%), distal tibia (6%), pelvis (2%), sacrum (2%), and spine (3%) [2]. GCTB has a high local recurrence rate (median, 20%) [3], 2–9% of GCTBs develop lung metastases [4], and approximately 2.4% develop malignant transformation (secondary malignant GCTB) [5]. Histologically, GCTB comprises neoplastic mononuclear stromal cells with a monotonous appearance admixed with macrophages and osteoclast-like giant cells [1]. GCTB stromal cells highly express the receptor activation of nuclear factor-kappa ß (RANK) ligand (RANKL) [6,7]. In osteolytic tumors, similar to GCTB, massive bone resorption is triggered by the RANKL/RANK axis, which stimulates osteoclast-dependent and -independent pathways by activating intracellular mediators [8]. Patients with high carboxyterminal-crosslinked-telopeptide of type I collagen (s-CTX) (≥500 UI/mL), a marker of bone resorption, had significantly worse outcomes compared to those with low s-CTX (<500 UI/mL) [9]. It has also been reported that the median s-CTX was higher in patients with tumor sizes ≥5 cm [9].

Systemic inflammation plays an important role in the development, progression, and metastasis of malignant tumors [10]. Several serum systemic inflammatory markers have been reported to help predict the prognosis of patients with malignancy, including the neutrophil-lymphocyte ratio (NLR), modified Glasgow prognostic score (mGPS), prognostic nutritional index (PNI), lymphocyte-monocyte ratio (LMR), and platelet-lymphocyte ratio (PLR) [11]. These markers can be easily obtained using routine blood tests in patients with tumors [11]. Even in intermediate-grade GCTB, the NLR [12,13,14], PNI [15], and PLR score [13] have been reported to correlate with the local recurrence rate after surgery. Decreased levels of hemoglobin (Hb), which are often observed in patients with malignant tumors [16,17,18,19]; alkaline phosphatase (ALP), which reflects bone metabolic turnover, osteoblast activity, and osteogenesis in bone tissue [20,21,22,23]; and lactate dehydrogenase (LDH), which promotes energy metabolism in tumor cells [24,25], are also useful serum markers for predicting the prognosis of patients with malignant tumors.

This study aimed to investigate the correlation between serum systemic inflammatory markers, such as NLR, mGPS, PNI, LMR, PLR, and Hb, ALP, and LDH levels, and local recurrence in patients with GCTB.

## 2. Materials and Methods

**Figure 1:**
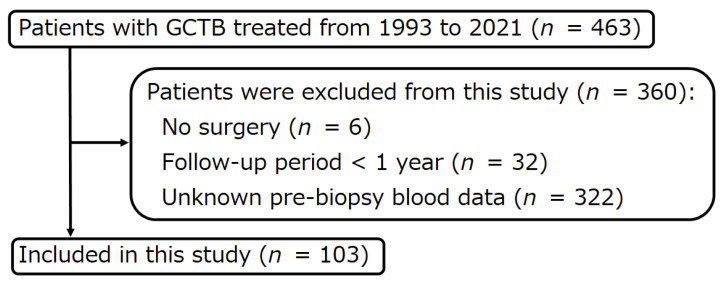
Flow diagram of patients with giant cell tumor of bone treated between 1993 and 2021.

Of the 463 patients with GCTB treated at the authors’ institutions between January 1993 and December 2021, 6 patients who did not undergo surgery, 32 patients with a postoperative follow-up of fewer than 12 months, and 322 patients with unknown pre-biopsy blood data were excluded. The remaining 103 patients were included in this retrospective study (Figure 1).

The following information was extracted from the medical records: sex, age, tumor location (the proximal femur, distal radius, hand, foot, and other sites were analyzed separately because of higher local recurrence rates in the proximal femur, distal radius, hand, and foot [26,27,28]), Campanacci stage [2], pathological fractures at presentation, pre- and postoperative denosumab administration, primary or recurrent tumor, pre-biopsy blood data (C-reactive protein (CRP) (mg/L), neutrophil count (×10^9^/L), lymphocyte count (×10^9^/L), monocyte count (×10^9^/L), platelet count (×10^9^/L), Hb (g/L), ALP (U/L), LDH (U/L), NLR, mGPS, PNI, LMR, PLR score, local recurrence, lung metastasis, postoperative follow-up period, and oncological outcomes (Table 1).

The NLR, PNI, LMR, and PLR score were calculated according to the following formulas: NLR—neutrophil/lymphocyte counts, PNI—albumin (g/L) + 5 × total lymphocyte counts per liter, LMR—lymphocyte/monocyte counts, and PLR—platelet/lymphocyte counts [11,15]. For mGPS, CRP ≤ 10 mg/L and albumin ≥ 35 g/L were defined as 0 points, CRP > 10 mg/L as 1 point, and CRP > 10 mg/L and albumin < 35 g/L as 2 points [29,30].

### 2.1. Curettage

Curettage was indicated for GCTB with moderate cortical thinning, well-maintained bone structure, and simple pathological fractures [26,31,32]. Curettage was performed in 63 patients through a large cortical bone window with a sharp curette that allowed the resection of all visible tumors [26,31,32]. The cavity was then curetted using a high-speed burr and washed with saline to resect all the tumors [26,31,32]. In 35 patients, phenol was applied to the border of the cavity with cotton-tipped applicators and diluted with alcohol. Cryosurgery using liquid nitrogen spray was performed in six patients, and ablation using an argon beam coagulator was performed in four patients. The tumor cavity was then filled with polymethylmethacrylate (PMMA) bone cement alone (27 patients), PMMA and bone allograft (16 patients), bone allograft alone (8 patients), or hydroxyapatite graft alone (12 patients).

### 2.2. En Bloc Resection

En bloc resection (resection of a large bulky tumor and its attached normal structures, virtually without dissection) was performed in 40 patients with large tumors with soft tissue extension, pathological fractures with joint involvement, complex fractures, and dispensable bones, such as the proximal fibula and distal ulna [32]. No reconstruction was performed in 5 patients, reconstruction with allograft in 11 patients, reconstruction with a prosthesis in 19 patients, and allograft prosthetic composite in 5 patients.

### 2.3. Denosumab

Denosumab (a fully human monoclonal antibody that inhibits RANKL) was approved by the US Food and Drug Administration in 2013 because of its reported efficacy and safety [33]. It was also reported that denosumab had the effect of down-staging to less invasive surgery [34]. Currently, denosumab treatment is indicated for GCTB that is inoperable or where resection will result in severe dysfunction [33]. Denosumab was administered in patients with minimal residual periarticular and subchondral bone, large extraskeletal lesions (Campanacci stage 3), and pathological fractures that made joint preservation difficult. Denosumab was administered particularly in patients with GCTB in the distal radius (for downstaging the tumor) because tumors at this site are aggressive, and their resection is associated with poor functional results [28]. Preoperatively, denosumab (120 mg) was administered subcutaneously once a week for 1 month and then once a month for 3–29 months. Postoperatively, it was administered at the same dose once a month for 1–6 months.

Routine follow-up examinations were performed every 4 months for the first 2 years, every 6 months for the next 3 years, and annually thereafter. Follow-up evaluations included radiography of the tumor area and computed tomography of the chest. Local recurrence and lung metastases were also recorded.

The difference between the two independent samples was statistically analyzed using the Mann-Whitney U test for nonparametric analyses. Local recurrence-free survival was defined as the time from surgery to local recurrence or last follow-up. To examine the correlation between each variable and local recurrence-free survival, univariate analysis was performed using Cox proportional hazards regression analysis and Kaplan-Meier survival analysis (a log-rank test), and multivariate analysis was performed using Cox proportional hazards regression analysis. Statistical significance was set at *p* < 0.05. All analyses were performed using JMP 14 (SAS Institute Inc., Cary, NC, USA) and IBM SPSS (version 28.0; IBM Co., Armonk, NY, USA).

The study was conducted in accordance with the Declaration of Helsinki and approved by the individual Institutional Review Board (or Ethics Committee) of IRCCS Istituto Ortopedico Rizzoli (protocol code 0008286 and date of approval 15 March 2016) and Nara Medical University (protocol code 2833 and date of approval 27 November 2020).

## 3. Results

Local recurrence was observed in 30 (29.1%) patients, with a median time from surgery to local recurrence of 16 (interquartile range (IQR), 10.8–23.3) months. Lung metastases were observed in nine (8.7%) patients, and the median time from the date of diagnosis of GCTB to lung metastasis was 11 (IQR, 0–35) months. The median postoperative follow-up period was 66 (IQR, 9.5–543) months. Malignant transformation was observed in three patients, and the times from surgery to malignant transformation were 20, 49, and 137 months, respectively. Two of these patients survived after resection of the primary tumor and adjuvant chemotherapy; the other had lung metastasis and died of the tumor after chemotherapy. With respect to the oncological results, 66 patients remained disease free, and 26 patients had no evidence of disease after treatment for local recurrence. Three patients had no evidence of disease after lung metastasis treatment, and five were alive with lung metastases. One patient died of this disease. Two patients died from another disease.

**Figure 2:**
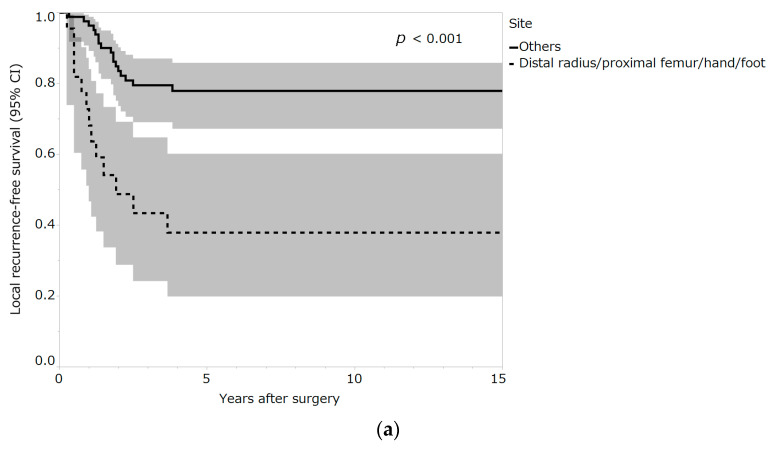

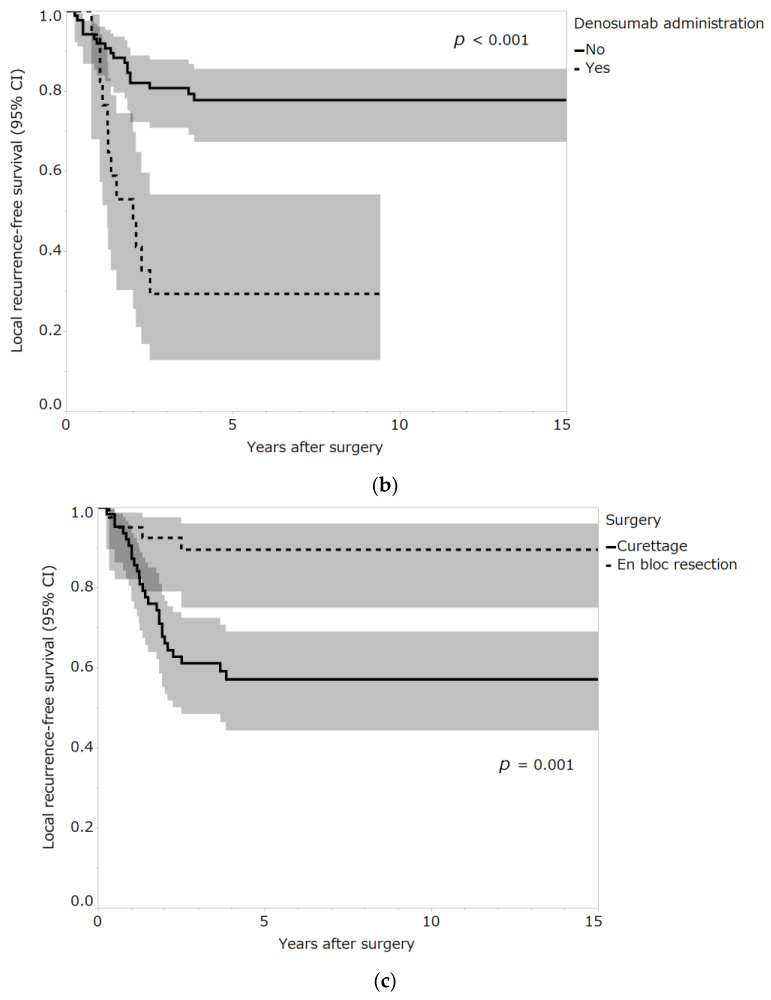
(**a**) Local recurrence—free survival rates of patients by tumor site. Shading around the curves represents the 95% confidence interval (CI). (**b**) Local recurrence—free survival rates of patients by denosumab administration. Shading around the curves represents the 95% CI. (**c**) Local recurrence—free survival rates of patients by the surgical method. Shading around the curves represents the 95% CI.

On univariate analysis, patients with GCTB of the distal radius/proximal femur/hand/foot presented a higher risk for local recurrence compared with patients who had GCTB at another site (hazard ratio (HR) = 4.16; 95% confidence interval (CI), 2.01–8.59; *p* < 0.001; Table 2) (Figure 2a).

Patients treated with denosumab presented a higher risk of local recurrence than patients treated without denosumab (HR = 4.42; 95% CI, 2.11–9.22; *p* < 0.001; Table 2) (Figure 2b). Patients treated with en bloc resection had a lower risk of local recurrence than those treated with curettage (HR = 0.21; 95% CI, 0.07–0.60; *p* < 0.001; Table 2) (Figure 2c). Elevated LDH levels were associated with a decreased risk of local recurrence (HR = 0.994; 95% CI, 0.990–0.998; *p* = 0.009; Table 2). Stepwise multivariate analysis was performed using the four clinical variables that showed significant differences (Table 2). Multivariate analysis showed that distal radius/proximal femur/hand/foot, denosumab treatment, and curettage were independent risk factors for unfavorable local recurrence-free survival (HR 4.36 [95% CI, 2.03–9.35], *p* < 0.001; HR 2.27 [95% CI, 1.02–5.06], *p* = 0.046; and HR 0.22 [95% CI, 0.07–0.68], *p* = 0.008, respectively) (Table 3).

**Figure 3:**
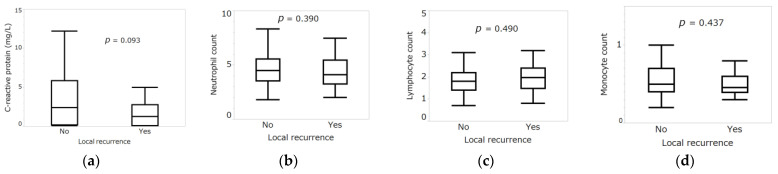

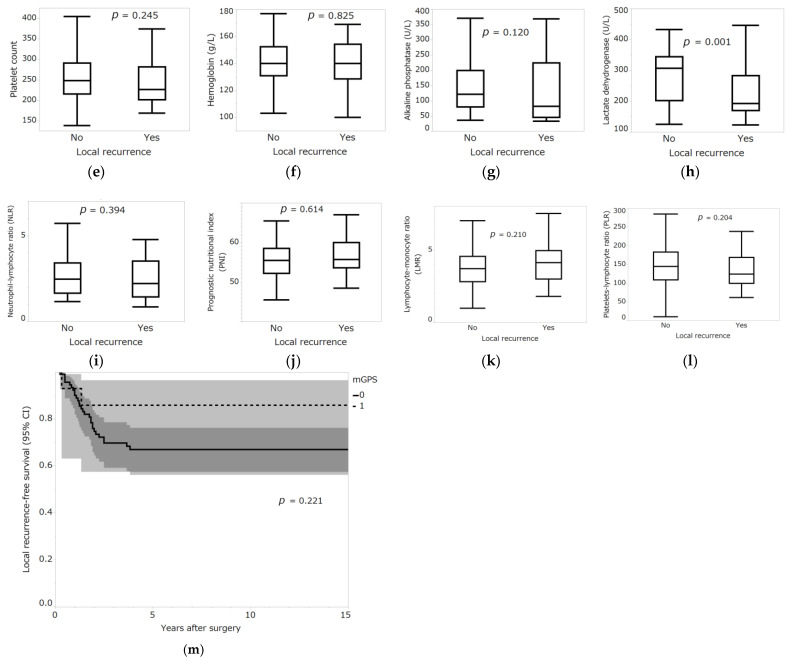
(**a**) Mann-Whitney U test comparing C-reactive protein with and without local recurrence. (**b**) Mann-Whitney U test comparing neutrophil count (×10^9^/L) with and without local recurrence. (**c**) Mann-Whitney U test comparing lymphocyte count (×10^9^/L) with and without local recurrence. (**d**) Mann-Whitney U test comparing monocyte count (×10^9^/L) with and without local recurrence. (**e**) Mann-Whitney U test comparing platelet count (×10^9^/L) with and without local recurrence. (**f**) Mann-Whitney U test comparing hemoglobin with and without local recurrence. (**g**) Mann-Whitney U test comparing alkaline phosphatase with and without local recurrence. (**h**) Mann-Whitney U test comparing lactate dehydrogenase with and without local recurrence. (**i**) Mann-Whitney U test comparing neutrophil-lymphocyte ratio (NLR) with and without local recurrence. (**j**) Mann-Whitney U test comparing prognostic nutritional index (PNI) with and without local recurrence. (**k**) Mann-Whitney U test comparing lymphocyte-monocyte ratio (LMR) with and without local recurrence. (**l**) Mann-Whitney U test comparing platelet–lymphocyte ratio (PLR) with and without local recurrence. (**m**) Local recurrence-free survival rates of patients by modified Glasgow prognostic score (mGPS). Shading around the curves represents the 95% confidence intervals (CIs).

Thus, no correlation was found between any inflammatory marker and local recurrence (Table 2, Table 3 and Table 4) (Figure 3).

## 4. Discussion

Several cytokines, such as interleukins (ILs), vascular endothelial growth factor, and tumor necrosis alpha, stimulate the production of granulocytes and platelets during tumor growth [35]. In addition, serum albumin and lymphocytes have also been found to exert anti-tumor effects by enhancing the immune response against tumors [36,37]. Thus, the extent of the inflammatory response has been investigated in several tumors using platelets, neutrophils, lymphocytes, monocytes, and albumin and has been found to predict prognosis and therapeutic response [38]. In addition, NLR, PNI, PLR, and LMR score, a combination of the above factors, have been used to assess the prognosis of different tumor types. In GCTB, associations between NLR [12,13,14], PNI [15], and PLR [13] and local recurrence rates have been reported. However, in the present study, none of the inflammatory markers (NLR, PLR, LMR, or PNI score) correlated with local recurrence rates in patients with GCTB.

Based on the result of this study, the site of the distal radius/proximal femur/hand/foot, denosumab treatment, and curettage were independent risk factors for unfavorable local recurrence. The high local recurrence rate in the distal radius is attributed to the fact that the bone at this site is relatively fragile, and its proximity to the carpal and ulnar bones makes adequate curettage difficult [39,40]. The high local recurrence rate in the hands and feet is attributed to the fact that the fenestration is small as the bone is small, making it difficult to secure a sufficient field of view and perform adequate curettage [27]. The high local recurrence rate in the proximal femur is due to insufficient curettage because of fear of femoral head necrosis and fracture [41]. Preoperative denosumab treatment increases the local recurrence rate after curettage because preoperative administration of denosumab causes osteosclerosis, making curettage and identifying the extent of the tumor difficult, leading to the reactivation of giant cell tumor cells hidden in osteosclerotic lesions after denosumab discontinuation [42,43,44,45]. Concerning the surgical method, curettage can preserve the joint and improve postoperative function, but compared to en bloc resection, the tumor is more likely to be left behind, and the local recurrence rate is higher [41].

Cancer-related systemic inflammatory responses are often associated with an increase in the number of circulating neutrophils. Neutrophils secrete cytokines and chemokines that play important roles in cancer progression [46]. In contrast, lymphocytes can promote cytotoxic immune responses against cancer [47,48]. The NLR is used to assess inflammatory and immune status. A high NLR is associated with decreased survival in patients with colorectal, laryngeal, lung, endometrial, head and neck, and ovarian cancers [49,50,51,52,53,54,55].

Yapar et al. investigated the correlation between pretreatment NLR and local recurrence in 96 patients with GCTB. On multivariate analysis, they reported that NLR ≥ 2.25 was an independent poor prognostic factor for local recurrence-free survival [12]. Li et al. investigated the association between pretreatment NLR and disease-free survival in 129 patients with spinal GCTB. On multivariate analysis, NLR > 2.70 was an independent poor prognostic factor for disease-free survival [13]. Chen et al. investigated the association between the pretreatment NLR and local recurrence in 163 patients with GCTB of the extremities. On multivariate analysis, NLR > 2.32 was an independent poor prognostic factor for local recurrence-free survival [14]. In contrast, Liang et al. reported no correlation between pretreatment NLR and local recurrence in 105 patients with GCTB [15]. The present study showed no correlation between pretreatment NLR and local recurrence or distant metastasis. The studies by Yapar et al. and Chen et al. did not investigate factors strongly associated with local recurrence, such as tumor site and denosumab treatment, which may have led to different results from our study [12,14].

Albumin is an acute-phase protein whose levels decrease in response to inflammation. In addition, low albumin levels reflect malnutrition due to cancer and negatively impact the prognosis. mGPS, introduced by Forrest et al. and modified by McMillan et al., is a well-known inflammation-related marker [30,56]. The mGPS is defined as CRP ≤ 10 mg/L and albumin ≥ 35 g/L at 0 points, CRP > 10 mg/L at 1 point, CRP > 10 mg/L, and albumin < 35 g/L at 2 points [29,30]. In other words, a high mGPS reflects both active systemic inflammation (elevated CRP level) and low nutritional status (hypoalbuminemia). According to McMillan’s review [29], this score has been used in over 60 studies (*n* = 30,000 patients) in 13 different countries across several tumors and treatment types. It has been found to reproducibly predict cancer mortality independent of disease severity. The GPS may predict prognosis in relation to weight and muscle loss, malnutrition, comorbidity, and treatment toxicity [29]. However, in the present study, we found no correlation between pretreatment mGPS and local recurrence or distant metastasis in patients with GCTB.

The PNI is another well-known albumin-related prognostic marker [36]. The PNI score is calculated as “albumin level (g/L) + 0.005 × lymphocytes”; decreased PNI score correlates with poor prognosis in patients with advanced-stage cancer treated with immune checkpoint inhibitors and in patients with pancreatic, renal, and esophageal cancers [57,58,59,60]. Liang et al. investigated the association between pretreatment PNI score and local recurrence rates in 105 patients with GCTB. Multivariate analysis showed that a PNI score < 48.6 was an independent poor prognostic factor for local recurrence-free survival [15]. This study found no correlation between the pretreatment PNI score and local recurrence or distant metastasis. Liang et al. did not investigate factors strongly associated with local recurrence, such as tumor site and denosumab treatment, which may have led to different results from our study [15].

Monocytes play an essential role in tumor progression in the tumor microenvironment. Monocytes differentiate into tumor-associated macrophages, which contribute to tumor invasion and metastasis. An increased serum monocyte count reflects tumor-associated macrophage activity. A lower LMR, indicating lower lymphocyte counts and higher monocyte counts, may reflect an active inflammatory state. Low levels of LMR are associated with decreased survival in patients with lung and pancreatic cancers [61,62]. Yapar et al. reported no correlation between pretreatment LMR and local recurrence in 96 patients with GCTB [12]. Li et al. reported no correlation between pretreatment LMR and disease-free survival in 129 patients with spinal GCTB [13]. In the present study, we found no correlation between pretreatment LMR and local recurrence or distant metastasis, confirming the previous data published in the literature.

Thrombocytopenia is often observed in patients with solid tumors and chronic inflammation [63,64]. In the tumor microenvironment, platelets can promote tumorigenesis by enhancing angiogenesis by releasing angiogenic proteins, such as vascular epidermal growth factor, and transforming growth factor-beta. Platelet-derived growth factors also play an important role in promoting tumor growth and invasion. In addition, the cytokines and chemokines produced by platelets promote cancer-related inflammation. The PLR has been a well-known prognostic marker. A high PLR reflects an increased platelet count and a decreased lymphocyte count. High PLR is associated with reduced survival in patients with laryngeal, lung, endometrial, and ovarian cancers [50,51,52,55,65]. Indeed, in GCTB, vascular epidermal growth factor receptors support RANKL-induced osteoclastogenesis, and vascular epidermal growth factor receptors are upregulated in patients with large and recurrent GCTB [66,67]. Therefore, the multitarget tyrosine kinase inhibitor “Lenvatinib,” which acts through the inhibition of vascular endothelial growth factor receptors, fibroblast growth factor receptors, platelet-derived growth factor receptor, proto-oncogene receptor tyrosine kinase, and rearranged during transfection kinase, has been suggested to be effective against GCTB [68].

Li et al. investigated the association between pretreatment PLR and disease-free survival in 129 patients with spinal GCTB. Multivariate analysis showed that PLR > 215.8 was an independent poor prognostic factor for disease-free survival [13]. In contrast, Yapar et al. reported no correlation between pretreatment PLR and local recurrence in 96 patients with GCTB [12]. Chen et al. reported no correlation between pretreatment PLR and local recurrence in 163 patients with GCTB of the extremities [14]. Liang et al. reported no correlation between pretreatment PLR and local recurrence in 105 patients with GCTB [15]. We also found no correlation between pretreatment PLR and local recurrence or distant metastasis in the present study.

A decreased Hb level is the most observed hematological abnormality in patients with cancer. It is induced by the direct or indirect effects of malignancy or its treatment [69]. The mechanisms underlying Hb degradation in cancer are complex. Blood loss, hemolysis, bone marrow infiltration, and nutritional deficiencies may cause Hb decline. In addition, cancer-stimulated production of inflammatory cytokines (e.g., tumor necrosis factor-α, IL-1, IL-6, and interferon-γ) inhibits erythropoiesis and leads to Hb decline [70,71]. Lower pretreatment Hb levels are associated with decreased survival in patients with lung, prostate, bladder, and ovarian cancers [16,17,18,19]. However, Yapar et al. reported no correlation between pretreatment Hb levels and local recurrence rates in 96 patients with GCTB [12]. Likewise, the present study showed no correlation between pretreatment Hb levels and local recurrence or distant metastasis.

The prognostic value of ALP has been shown in patients with various solid malignancies with bone metastases and osteosarcoma [20,21,22,23]. Once cancer begins metastasizing to the bone, ALP reflects bone metabolic turnover, osteoblast activity, and osteoid formation in the adjacent bone tissue [23]. Thus, ALP is an indicator of the metastatic bone tumor burden. However, Chen et al. reported no correlation between pretreatment ALP level and local recurrence rate in 163 patients with GCTB in the extremities [14]. We also found no correlation between pretreatment ALP level and local recurrence or distant metastasis in the present study.

The energy metabolism of tumor cells differs from that of normal cells and is considered one of the hallmarks of cancer, consisting mainly of aerobic glycolysis, fatty acid oxidation, and glutaminolysis. Since Warburg observed that cancer cells prefer aerobic glycolysis, even under normoxic conditions, and maintain the high growth rates of cancer cells, 100 years have passed. LDH is a critical enzyme that promotes this phenomenon. Furthermore, high LDH levels are a marker of poor prognosis in patients with lung cancer [24,25]. However, this study found no correlation between pretreatment LDH levels and local recurrence or distant metastasis in patients with GCTB.

This study has some limitations. First, there is the possibility of a type 2 error owing to the small number of cases. If the number of cases increases, there is a possibility that factors with significant differences will emerge. A multivariate analysis with a larger number of cases is required. Second, it is possible that the time of blood sampling, whether the patient has fasted, medical history, and comorbidities affected the inflammatory markers, and that effect was not considered.

## 5. Conclusions

In this study, inflammatory markers, such as NLR, mGPS, PLR, LMR, PNI, and Hb, ALP, and LDH levels, did not correlate with local recurrence in patients with GCTB. However, due to the small number of patients included in this study, this result should be re-evaluated in a multicenter study with a larger sample size.

## Figures and Tables

**Table 1:** Patient characteristics included in this study.

Variable (*n* = 103)	No. of Patients
Sex	
Male	54 (52.4%)
Female	49 (47.6%)
Age (years)	Median, 33.6 (IQR, 23.6–44.4)
Site	
Distal radius	13 (12.6%)
Proximal femur	6 (5.8%)
Distal femur	36 (35.0%)
Proximal tibia	27 (26.2%)
Proximal fibula	4 (3.9%)
Proximal humerus	4 (3.9%)
Hand and foot	3 (2.9%)
Others *	10 (9.7%)
Campanacci classification	
Stage I	0
Stage II	63 (61.2%)
Stage III	40 (38.8%)
Lung metastasis at presentation	
No	99 (96.1%)
Yes	4 (3.9%)
Pathological fracture at presentation	
No	88 (85.4%)
Yes	15 (14.6%)
Denosumab administration	
No	86 (83.5%)
Yes	17 (16.5%)
Previous surgery	
No	84 (81.6%)
Yes	19 (18.4%)
Surgery	
Curettage	63 (61.2%)
En bloc resection	40 (38.8%)
C-reactive protein (mg/L)	Median, 2 (IQR, 0–5.3)
Neutrophil count (×10^9^/L)	Median, 4.3 (IQR, 3.4–5.6)
Lymphocyte count (×10^9^/L)	Median, 1.9 (IQR, 1.4–2.3)
Monocyte count (×10^9^/L)	Median, 0.5 (IQR, 0.4–0.6)
Platelet count (×10^9^/L)	Median, 244 (IQR, 207–288)
Serum hemoglobin (g/L)	Median, 140 (IQR, 130–153)
Alkaline phosphatase (U/L)	Median, 106 (IQR, 63–200)
Lactate dehydrogenase (U/L)	Median, 281 (IQR, 180–334)
Neutrophil-lymphocyte ratio (NLR)	Median, 2.41 (IQR, 1.59–3.42)
Modified Glasgow prognostic score (mGPS)	
0	89 (86.4%)
1	14 (13.6%)
2	0
Prognostic nutritional index (PNI)	Median, 55.5 (IQR, 53–58.7)
Lymphocyte-monocyte ratio (LMR)	Median, 3.83 (IQR, 2.86–4.67)
Platelet-lymphocyte ratio (PLR)	Median, 135.5 (IQR, 105.6–178.6)
Local recurrence	
None	73 (70.9%)
≥1	30 (29.1%)
Lung metastasis	
None	94 (91.3%)
≥1	9 (8.7%)
Follow-up (months)	Median, 66 (IQR, 9.5–543)

IQR—interquartile range. * Other sites include the distal ulna (3), distal tibia (2), distal humerus (1), calcaneus (1), proximal ulna (1), vertebra (1), and ischium (1).

**Table 2:** Univariate Cox regression analysis of local recurrence-free survival.

Variable (*n* = 103)	No. of Patients	Hazard Ratio (95% CI)	*p*-Value
Sex			0.307
Male	54	1	
Female	49	1.45 (0.71–2.98)	
Age (years)	103	0.98 (0.96–1.01)	0.195
Site			<0.001 *
Distal radius/proximal femur/hand/foot	22	4.16 (2.01–8.59)	
Others	81	1	
Campanacci classification			0.806
Stages I and II	63	1	
Stage III	40	0.91 (0.43–1.92)	
Pathological fracture at presentation			0.962
No	88	1	
Yes	15	0.97 (0.34–2.79)	
Denosumab administration			<0.001 *
No	86	1	
Yes	17	4.42 (2.11–9.22)	
Previous surgery			0.366
No	84	1	
Yes	19	1.48 (0.63–3.45)	
Surgery			<0.001 *
Curettage	63	1	
En bloc resection	40	0.21 (0.07–0.60)	
C-reactive protein (mg/L)	103	0.99 (0.97–1.01)	0.546
Neutrophil count (×10^9^/L)	103	0.96 (0.85–1.09)	0.553
Lymphocyte count (×10^9^/L)	103	0.97 (0.79–1.20)	0.796
Monocyte count (×10^9^/L)	103	0.42 (0.05–3.34)	0.410
Platelet count (×10^9^/L)	103	1.00 (0.99–1.00)	0.308
Serum hemoglobin (g/L)	103	1.00 (0.97–1.02)	0.637
Alkaline phosphatase (U/L)	103	1.00 (0.99–1.00)	0.526
Lactate dehydrogenase (U/L)	103	0.994 (0.990–0.998)	0.009 *
Neutrophil-lymphocyte ratio (NLR)	103	0.96 (0.81–1.14)	0.616
Modified Glasgow prognostic score (mGPS)			0.235
0	89	1	
1	14	0.42 (0.10–1.76)	
Prognostic nutritional index (PNI)	103	0.99 (0.96–1.03)	0.740
Lymphocyte-monocyte ratio (LMR)	103	1.09 (0.89–1.35)	0.401
Platelet-lymphocyte ratio (PLR)	103	1.00 (0.99–1.00)	0.411

* Statistically significant.

**Table 3:** Multivariate Cox regression analysis of local recurrence-free survival.

Variable	Hazard Ratio (95% CI)	*p*-Value
Site		
Distal radius/proximal femur/hand/foot versus others	4.36 (2.03–9.35)	<0.001 *
Denosumab administration		
Yes versus no	2.27 (1.02–5.06)	0.046 *
Surgery		
En bloc resection versus curettage	0.22 (0.07–0.68)	0.008 *

* Statistically significant.

**Table 4:** Association between inflammatory markers and local recurrence.

Variable	Local Recurrence: No (Median (IQR))	Local Recurrence: Yes (Median (IQR))	*p*-Value
C-reactive protein (mg/L)	2.4 (0.2–5.85)	1.25 (0.1–2.78)	0.093
Neutrophil count (×10^9^/L)	4.5 (3.55–5.55)	4.1 (3.25–5.45)	0.390
Lymphocyte count (×10^9^/L)	2 (1.55–2.5)	2.25 (1.58–3.05)	0.490
Monocyte count (×10^9^/L)	0.5 (0.4–0.7)	0.46 (0.39–0.6)	0.437
Platelet count (×10^9^/L)	248 (215–291)	226 (201–282)	0.245
Hemoglobin (g/L)	140 (131–152.5)	140 (128.5–154.3)	0.825
Alkaline phosphatase (U/L)	122 (79.5–199.5)	82 (45.5–224)	0.120
Lactate dehydrogenase (U/L)	307 (202–344.5)	192 (169.5–283.3)	0.001 *
Neutrophil–lymphocyte ratio (NLR)	2.48 (1.65–3.41)	2.21 (1.44–3.53)	0.394
Prognostic nutritional index (PNI)	55.5 (52.3–58.6)	55.8 (53.6–60)	0.614
Lymphocyte-monocyte ratio (LMR)	3.71 (2.82–4.55)	4.13 (3–4.95)	0.210
Platelet-lymphocyte ratio (PLR)	144 (109–183)	124 (100–169)	0.204
Modified Glasgow prognostic score (mGPS)	No. of patients (*n* = 103)	5-year local recurrence-free survival (95% CI) (%)	*p*-value
0	89	66.8 (56–76.1)	0.221
1	14	85.7 (57.3–96.4)	

IQR—interquartile range; * Statistically significant.

## Data Availability

The datasets generated, analyzed, or both during the present study are not publicly available because of privacy issues but are available from the corresponding author upon reasonable request.
